# Prospective analysis of human leukocyte functional tests reveals metal sensitivity in patients with hip implant

**DOI:** 10.1186/1749-799X-8-12

**Published:** 2013-05-16

**Authors:** Csaba Vermes, József Kuzsner, Tamás Bárdos, Péter Than

**Affiliations:** 1 Department of Orthopedic Surgery, Institute of Musculoskeletal Surgery, University of Pécs Clinical Center, 1 Akác utca, Pécs 7632, Hungary

**Keywords:** Hip replacement, Metal allergy, Hypersensitivity, Leukocyte assay

## Abstract

**Background:**

The aim of the study was to examine the reactivity of peripheral human leukocytes to various metal ions prior and following hip replacement in order to investigate implant-induced metal sensitivity.

**Methods:**

Three patient groups were set up: (1) individuals without implants and no history of metal allergy (7 cases), (2) individuals without implants and known history of metal allergy (7 cases), and (3) patients undergoing cementless hip replacement (40 cases). Blood samples were taken in groups 1 and 2 at three different occasions; in group 3, prior and 3, 6, 12, 24, and 36 months after surgery. Peripheral leukocytes were separated and left either untreated or challenged with Ti, NiCl_2_, CoCl_2_, CrCl_3_, and phytohemagglutinin. Cell proliferation, cytokine release, and leukocyte migration inhibition assays were performed. Metal-induced reactivity was considered when all three assays showed significant change. Skin patch tests were also carried out.

**Results:**

Both skin patch tests and leukocyte functional tests were negative in group 1, and both were positive in group 2. In group 3, after 6 months, 12% of the patients showed reactivity to the tested metals except for NiCl_2_. Following the 36-month period, 18% of group three became sensitive to metals (including all the earlier 12%). In contrast, patch tests were negative at each time point in group 3.

**Conclusions:**

Orthopedic implant material may induce metal reactivity after implantation in a manner where susceptibility is yet to be elucidated. Leukocyte triple assay technique might be a useful tool to test implant material-related sensitivity.

## Background

Joint replacement has become a routine procedure, over 1 million performed annually worldwide. Nowadays, the predominant components of orthopedic implant materials are various metals. Due to continuous developing, the up-to-date metal alloy endoprostheses have great biomechanical and biocompatible properties. Implanted metals, however, undergo corrosion, and various degradation products are released including metal ions [1,2]. These molecules can activate the immune system as haptens by forming complexes with naturally occurring proteins. Metal–protein complexes are potential antigens inducing hypersensitivity responses (i.e., delayed type, type IV, cell-mediated adoptive reactions). Nickel is the most common metal sensitizer in humans followed by cobalt and chromium and in a lesser extent titanium also. Peripheral T lymphocytes get into contact with metals, and a subpopulation of these cells (T_H1_) become activated releasing different cytokines (tumor necrosis factor-α (TNF-α), interferon-γ (IFN-γ), interleukin (IL)-1, IL-2). This process recruits macrophages to the activating site, and a proinflammatory milieu develops. This may affect negatively the biocompatibility and osteointegration of joint implants [3-6].

Testing implant-related metal hypersensitivity is a specific task. Routinely, skin patch tests have been performed for decades [7-9]. In this case, however, the antigen-presenting cell is Langerhans cell in the skin, which is not predominant within the periprosthetic space. More likely, peripheral mononuclear cells (e.g., lymphocyte/monocytes) function as antigen-presenting cells around orthopedic implants. Thus, the development of various *in vitro* functional cell assays (proliferation, cytokine production, and migration inhibition tests) performed on peripheral leukocytes became necessary in order to gain insight into the implant-related metal hypersensitivity reactions [10-14]. Metal degradation products besides their prominent local effects, may cause remote problems, as metals and activated cell populations invade the human body [15-18].

We hypothesized that metallic components of hip implants can induce metal sensitivity in patients where metal allergy earlier was not present. Our aim was to examine the reactivity of human leukocytes to various metals before and after hip replacement and detect possible implant-induced metal reactivity/allergy in a prospective manner.

## Materials and methods

### Patient groups

Three patient groups were established: (1) age-matched patients without any implant and no history of metal allergy tested via skin patch test analysis (7 cases: 4 male, 3 female, average age: 59 years), (2) age-matched patients without any implant with a positive history of metal hypersensitivity analyzed by skin patch test (7 cases: 3 male, 4 female, average age: 62 years), and (3) patients undergoing cementless hip replacement with no history of metal allergy prior the surgery (40 cases: 20 male, 20 female, average age: 61 years) analyzed by skin patch testing and leukocyte functional testing (described later). Informed consent was obtained from all subjects after institutional review board review and approval. Implants used in the study are comprised of a titanium-6%aluminum-4%vanadium alloy stem with a cobalt–chromium femoral head, an ultrahigh molecular weight polyethylene acetabular liner in a titanium-6%aluminum-4%vanadium alloy shell, and the patients had no other implant.

### Sample collection and cell cultures

Peripheral blood mononuclear cells (PBMCs) were isolated from 20 ml of peripheral blood from peripheral venipuncture using density gradient separation (Ficoll-isopaque, Pharmacia, Piscataway, NJ, USA). Serum was also collected. Ficoll-separated mononuclear cells consist of mainly lymphocytes. Allergen-challenged lymphocytes respond with increased proliferation, cytokine production, and migration inhibition. Leukocytes in all assays were incubated with Dulbecco’s modified Eagle’s medium and 10% autologous serum [19].

### Treatment of cells

Leukocytes in all assays were incubated with either no metal (plain media) as a negative control, 0.01 mg/ml phytohemagglutinin (PHA) as a positive control, or metal for 5 days. Metals used in the *in vitro* studies were the more prevalent implant–alloy metals. The following concentrations were applied: 0.1 mM CrCl_3_, 0.1 mM NiCl_2_, 0.1 mM CoCl_2_ (Sigma, St. Louis, MO, USA) and approximately 0.01 mM titanium (using culture medium from incubated titanium beads) according to earlier recommendations [19,20]. The lower concentration of titanium is due to the fact that this alloy has insoluble property at physiologic pH and its subsequent inability to form ions in solution; nonetheless, this would be expected *in vivo* also [21].

### Proliferation assay (lymphocyte transformation tests)

We used standard lymphocyte transformation testing (LTT) protocol of mononuclear cells to measure lymphocyte proliferation in a 96-well plate system, [^3^H]-thymidine was added (1 mCi/culture well) during the last 12 h of incubation after 4 1/ 2 days of treatment. At day 5, [^3^H]-thymidine uptake was measured using liquid scintillation beta plate analysis (Wallac Gatesburg, MD, USA). The amount of [^3^H]-thymidine incorporation for each metal treatment was compared to the non-treated control resulting in a ratio, referred here as proliferation rate. All the assays were performed in triplicates. The time lag, associated with *in vivo* lymphocyte proliferation in a delayed type hypersensitivity response, was simulated *in vitro* for 5 days of incubation [10].

### Cytokine analysis

Cytokine concentrations in supernatants of leukocyte cultures were obtained after 48 h of incubation following treatments. Levels of IFN-γ and tumor TNF-α were measured by enzyme-linked immunosorbent assays in 96-well plates following the manufacturer’s instructions (assay range from 0.5 to 32 pg/ml, R&D Systems, Minneapolis, MN, USA and Sanquin, Amsterdam, Netherlands, all supplied by Biotest Hungary Ltd., Budapest, Hungary) [20]. All the assays were performed in triplicates.

### Leukocyte migration inhibition tests

The migratory capacity of PBMCs was assessed using a modified Boyden chamber technique. Briefly, 4 × 10^5^ cells were put in culture medium consisting 10% autologous serum and placed in the upper buffer chamber of a 24-well modified Boyden chamber (FluoroBok, BD Biosciences, Franklin Lakes, NJ, USA). The upper chamber was placed in the lower chamber containing plain medium or culture medium supplemented with PHA or metal treatments for 48 h at 37°C. Cells were then counted in both chambers using a microscope to determine the number of migrated cells [10,11]. All the assays were performed in triplicates by one observer (C.V.).

### Skin patch testing

Patch testing was performed using Finn chambers (8 mm) on Scanpor tape (Almirall Hermal, Reinbek, Germany). Nickel sulfate, cobalt chloride, chromium chloride, and titanium oxalate were tested in petrolatum in concentrations of 5%, 1%, and 0.5%, respectively, according to international standards. The patch tests were applied to the upper back and were occluded for 48 h. Readings were done on day 2, day 3, or day 4, and day 7 according to the recommendations from the International Contact Dermatitis Research Group. Homogeneous redness and infiltration in the entire test area were scored as a 1+ reaction. Homogeneous redness, infiltration, and vesicles in the test area were scored as a 2+ reaction, and homogeneous redness, infiltration, and coalescing vesicles in the test area were scored as a 3+ reaction. A 1+, 2+, or 3+ reading was interpreted as a positive response. An irritant response, a doubtful, or a negative reading was interpreted as a negative response [22].

### Statistical analysis

First, the distribution of the data was assessed (Kolmogorov–Smirnov test). Normally distributed data (LTT analysis) were subjected to statistical analysis using Student’s *t* tests. Student’s *t* tests for independent samples with unequal or equal variances were used to test equality of the mean values at a 95% confidence interval (*p* < 0.05). In case of not normally distributed samples (cytokine and migration inhibition assays) the Kruskall–Wallis non-parametric analysis of variance test was used. Subsequently, the Wilcoxon–Mann–Whitney test was used when the Kruskall–Wallis test showed significant change. Treatment-specific responders were selected based on a statistically significant 1.5 fold change in the selected assay (*p* < 0.05) [19].

## Results

### Proliferation assay

**Figure 1:**
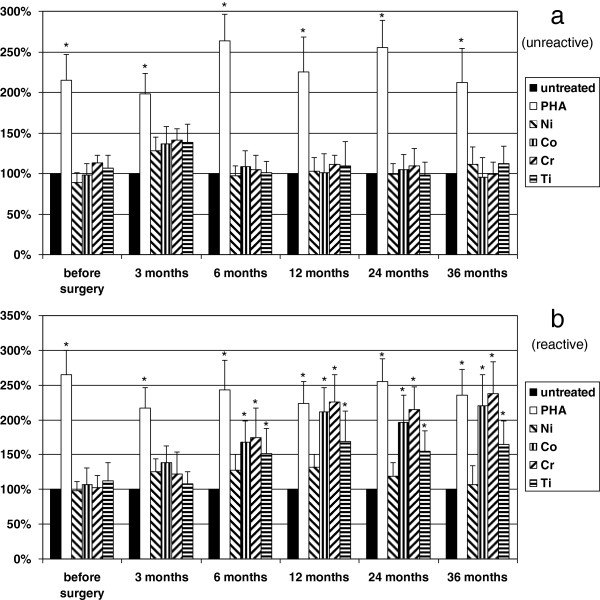
**The effect of various metals and PHA on the proliferation rate of patients’ leukocytes.** The effect of various metals (Ti 0.01 mM, Co 0.1 mM, Cr 0.1 mM, Ni 0.1 mM) and PHA (0.1 mg/ml) on the proliferation rate of patients’ leukocytes in group 3 is shown. Cells were untreated or treated as indicated. (**a**) (unreactive), it is clearly demonstrated that metal challenge did not influence cellular proliferation before and after the surgery. (**b**) (reactive), however, Co, Cr and Ti increased the proliferation rate significantly 6 months after surgery in 25% (10 patients) of group 3, which was increased up to 35% (14 patients) after 36 months, distinguishing a subgroup referred here as metal sensitive patients in group 3. Positive control PHA increased proliferation significantly in each experiment. Columns represent means + SD, level of significance is shown, ^*^p < 0.05. Note that Ni did not influence proliferation significantly.

Positive control PHA induced significantly (*p* < 0.05) increased proliferation rate in all patient groups along the study. In group 1, among the metals tested, only Ni stimulated cellular proliferation; however, it was not significant (*p* > 0.05). In group 2, where patients were selected based on positive skin patch metal allergy tests for all Ni, Co, Cr, and Ti, in the presence of these metals, proliferation rate was significantly (*p* < 0.05) elevated as expected. In group 3, before the surgery, none of the samples responded to metal challenge. After 6 months, 25% (*n* = 10) of the patients showed significantly higher proliferation rate in the presence of metals except for Ni. Even Ti, which was used in lower concentration, showed stimulation. Following the 36-month period, 35% (*n* = 14) become sensitive to metals (Figure 1). Interestingly, during the study, patch test was negative at each time point for all the metals tested in every patient.

### Cytokine production

**Figure 2:**
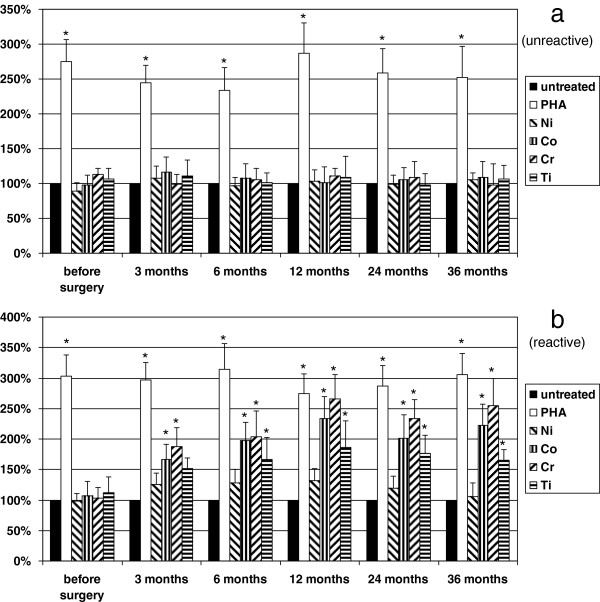
**The effect of various metals and PHA on IFN-γ production of patients’ leukocytes.** The effect of various metals (Ti 0.01 mM, Co 0.1 mM, Cr 0.1 mM, Ni 0.1 mM) and PHA (0.1 mg/ml) on IFN-γ production of patients’ leukocytes in group 3 is shown. Cells were untreated or treated as indicated. (**a**) (unreactive) it is clearly demonstrated that metal challenge did not influence significantly the cytokine release before and after the surgery. (**b**) (reactive) however, Co, Cr, and Ti increased the IFN-γ secretion significantly 3 months after surgery in 21% (8 patients) of group 3, which was increased up to 30% (12 patients) after 36 months, distinguishing a subgroup referred here as metal-sensitive patients in group 3. Positive control PHA increased IFN-γ production significantly in each experiment. Columns represent means + SD, level of significance is shown, ^*^p < 0.05. Note that Ni did not influence IFN-γ secretion significantly.

**Figure 3:**
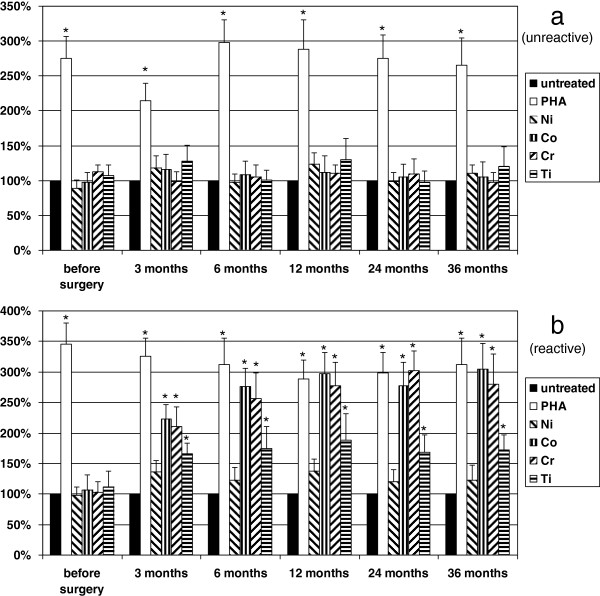
**The effect of various metals and PHA on TNF-α production of patients’ leukocytes.** The effect of various metals (Ti 0.01 mM, Co 0.1 mM, Cr 0.1 mM, Ni 0.1 mM) and PHA (0.1 mg/ml) on TNF-α production of patients’ leukocytes in group 3 is shown. Cells were untreated or treated as indicated. (**a**) (unreactive) it is clearly demonstrated that metal challenge did not influence significantly the cytokine release before and after the surgery. (**b**) (reactive) however, Co, Cr, and Ti increased the TNF-α secretion significantly 3 months after surgery in 21% (8 patients) of group 3, which was increased up to 30% (12 patients) after 36 months, distinguishing a subgroup referred here as metal-sensitive patients in group 3. Positive control PHA increased TNF-α production significantly in each experiment. Columns represent means + SD, level of significance is shown, ^*^p < 0.05. Note that Ni did not influence TNF-α secretion significantly.

Positive control PHA induced significantly (*p* < 0.05) increased secretion of IFN-γ and TNF-α in all patient groups along the study. In group 1, none of the metals tested stimulated cytokine production. In group 2, where patients were selected based on positive skin patch metal allergy tests for Ni, Co, Cr, and Ti, in the presence of these metals, cytokine release was significantly (*p* < 0.05) elevated in all patients. In group 3, before the surgery, none of the samples responded to metal challenge. After 3 months, 21% (*n* = 8) of the patients showed significantly higher secretion of IFN-γ and TNF-α in the presence of metals; Ni increased these parameters only modestly. Even Ti showed activation. Following the 36-month period, 30% (*n* = 12) of patients’ samples showed increased IFN-γ and TNF-α production *in vitro* (Figure 2 and 3). Again, patch test was negative at each time point for all the metals in every patient.

### Leukocyte migration inhibition

In group 1, metals did not modify the ability of leukocyte to actively migrate. In group 2, leukocytes showed inhibited migration in the presence of each metals used in the study. In group 3, before the surgery, none of the samples responded to metal challenge. After 6 months, 16% (*n* = 6) of the samples showed significantly altered migration of PBMCs in the presence of metals. Following the 36-month period, 23% (*n* = 9) of patients’ samples showed significantly changed leukocyte migration *in vitro*.

### Summary of the leukocyte assays

The results of functional leukocyte assays revealed reactivity to metals following hip replacement. Different assays, however, showed various fractions of group 3 which became sensitive to metals listed above. The reactive ratio of group 3 varied also by exposure time (3 months vs. 36 months). Thus, a patient was considered reactive when all three assays showed significant (*p* < 0.05) changes. Based on these criteria, 12% (*n* = 5) and 18% (*n* = 7) fraction were found reactive after 6 and 36 months, respectively, following surgery in group 3. The 12% portion was among the latter 18% fraction. All of the hip implants had good functions (average Harris Hip Score: 92), and none of them needed revision (no osteolysis on the X-ray) along the follow-up of the study.

## Discussion

Dermal hypersensitivity to different metals is well documented and affects approximately 10–12% of the general population. Nickel is the most common metal sensitizer in humans followed by cobalt and chromium and various responses have been reported to titanium as well, and cross-reactivity between nickel and cobalt is known. If metal sensitivity is known prior to implantation, specific implant components can be chosen [3,23-25].

The prevalence of metal sensitivity among patients with well-functioning and poorly functioning implants has been reported to be approximately 20% and 50%, respectively, as measured by dermal patch testing [3,7,8,23]. It was proposed that skin testing may not be the most appropriate method to test orthopedic implant-related metal sensitivity; thus, different *in vitro* functional assays performed on peripheral leukocytes have been developed and found even higher occurrence of metal sensitivity [9,14,21,26].

Using the combination of these assays, we found that 18% of patients became sensitive to metals 36 months after cementless hip replacement with well-functioning implants. Earlier, other studies using leukocyte assays have reported a larger fraction of patients to be sensitive to metals with well-functioning implants. The discrepancy is likely due to the different methods by which metal sensitivity was tested and the various time periods following surgery when the tests were carried out. The latter seems to be important because our study showed that the longer the exposure time to metals was (6 months vs. 36 months), the greater proportion of the cohort was found sensitive (12% vs. 18%). A patient was referred here to be reactive to a metal when all the tests applied in this study showed significant results at the maximum prospective follow-up time of 36 months. Many other studies, however, are not prospective, lacking the information about the prevalence of metal sensitivity in the cohort prior the operation, and mainly apply one method to analyze lymphocyte/leukocyte reactions at a long time period following surgery [19,21,26]. Taken together, we believe that combining the results of leukocyte assays is a stricter method to consider a sample reactive. This may lead to a more useful screening process to select patients with implant metal-induced sensitivity.

Patients with well-performing hip implants showed a higher incidence of metal reactivity, as determined by leukocyte assays than that of healthy controls. Interestingly, even Ti induced significant reactions, although in a lesser magnitude. Importantly, Ni did not induce leukocyte reaction along the study, which may be expected as implants applied did not contain Ni, showing that the measured reactivity to various metals were implant component specific. It seems that hip replacement procedure can sensitize patients’ leukocytes. Although the relationship between leukocyte assays and total hip arthroplasty clinical outcome remains undetermined, these results are consistent with the idea of individual susceptibility for hypersensitivity to metal components from implant degradation. The mechanism of such susceptibility and the clinical significance of these findings are yet to be determined because the long-term clinical performance of hip implants in a sensitized population and genetic background of individual responsiveness to metals are unknown to date.

Various immune responses to degradation products from implant materials can compromise the longevity of joint replacement [1,2,18,19]. Wear debris from bearing surfaces are generated continuously and phagocytosed by different cell types in the periimplant area inducing proinflammatory cascade in the periprosthetic space leading to the activation of osteoclasts and suppression of osteoblasts which in turn result in periprosthetic osteolysis, a process known as aseptic loosening of implants [1,27,28]. Lymphocytes can react with metals and mediate adoptive, cell-mediated delayed type hypersensitivity reaction. This seems to be T_H1_-type reaction as earlier studies proposed [19-21]. Our investigation also support this result as PBMCs, which contain approximately 85% lymphocytes, from patients with hip implants showed increased proliferation rate, T_H1_-type cytokine production and migration inhibition. Activated lymphocytes express mediators (e.g., receptor activator of nuclear factor kappa-B ligand) that ultimately leads to osteoclast activation. This mechanism alone may jeopardize the success of joint replacement and can worsen wear debris-induced aseptic implant loosening [29].

## Conclusions

Total joint replacement procedure may induce metal sensitivity in patients where metal allergy was not present earlier. This reaction can be revealed using specific *in vitro* cellular analyses. The result of combined leukocyte assays can be a useful tool to test implant material-related reactivity and may be superior to patch testing. These tests, however, are only capable of determining the presence of metal sensitivity following hip replacement, which provides the possibility only for secondary or tertiary prevention. The long-term significance of the present study by following the reactive patient group is that we will have data on the functions of these implants and compare metal sensitivity susceptible and non-susceptible patient groups in order to develop a diagnostic tool. In the future, one can envision a screening process where susceptibility for orthopedic implant-induced metal hypersensitivity can be determined before joint replacement providing possibility for primary prevention.

## Competing interests

The authors declare that they have no competing interest.

## Authors’ contribution

The experimental design, laboratory work, and preparation of the manuscript were done by CV. JK and TB assisted in the experimental processes, sample collections, and preparations. PT assisted in the experimental design and manuscript preparation and also provided patients for the study. All authors have read and approved the final manuscript.

## References

[B1] Jacobs JJ Roebuck KA Archibeck M Hallab NJ Glant TT Osteolysis: basic science Clin Orthop 2001 393 71 77 11764373 10.1097/00003086-200112000-00008

[B2] Zeng Y Feng W Metal allergy in patients with total hip replacement: a review J Int Med Res 2013 10.1177/0300060513476583 23569024

[B3] Hallab NJ Merritt K Jacobs JJ Metal sensitivity in patients with orthopedic implants J Bone Joint Surg Am 2001 83-A 428 436 11263649 10.2106/00004623-200103000-00017

[B4] Willert HG Buchorn GH Fayyazi A Flury R Windler M Köster G Lohmann CH Metal-on-metal bearings and hypersensitivity in patients with artificial hip joints. A clinical and histomorphological study J Bone Joint Surg Am 2005 87 28 36 15637030 10.2106/JBJS.A.02039pp

[B5] Park YS Moon YW Lim SJ Yang JM Ahn G Choi YL Early osteolysis following second-generation metal-on-metal hip replacement J Bone Joint Surg Am 2005 87 1515 1521 10.2106/JBJS.D.02641 15995119

[B6] Thyssen JP Jakobsen SS Engkilde K Johansen JD Soballe K Menne T The association between metal allergy, total hip arthroplasty, and revision Acta Orthop 2009 80 646 652 10.3109/17453670903487008 19995314 PMC2823320

[B7] Deutman R Mulder TJ Brian R Nater JP Metal sensitivity before and after total hip arthroplasty J Bone Joint Surg Am 1977 59 862 865 908716

[B8] Rooker GD Wilkinson JD Metal sensitivity in patients undergoing hip replacement. A prospective study J Bone Joint Surg Br 1980 62-B 502 505 7430234 10.1302/0301-620X.62B4.7430234

[B9] Granchi D Cenni E Giunti A Baldini N Metal hypersensitivity testing in patients undergoing joint replacement: a systematic review J Bone Joint Surg Br 2012 94 1126 1134 22844057 10.1302/0301-620X.94B8.28135

[B10] Hallab NJ Mikecz K Jacobs JJ A triple assay technique for the evaluation of metal-induced, delayed-type hypersensitivity responses in patients with or receiving total joint arthroplasty J Biomed Mater Res 2000 53 480 489 10.1002/1097-4636(200009)53:5<480::AID-JBM6>3.0.CO;2-B 10984695

[B11] Hallab N Jacobs JJ Black J Hypersensitivity to metallic biomaterials: a review of leukocyte migration inhibition assays Biomaterials 2000 21 1301 1314 10.1016/S0142-9612(99)00235-5 10850924

[B12] Hallab NJ Mikecz K Vermes C Skipor A Jacobs JJ Differential lymphocyte reactivity to serum-derived metal-protein complexes produced from cobalt-based and titanium-based implant alloy degradation J Biomed Mater Res 2001 56 427 436 10.1002/1097-4636(20010905)56:3<427::AID-JBM1112>3.0.CO;2-E 11372061

[B13] Thomas P Braathen LR Dorig M Aubock J Nestle F Werfel T Willert HG Increased metal allergy in patients with failed metal-on-metal hip arthroplasty and peri-implant T-lymphocytic inflammation Allergy 2009 64 1157 1165 10.1111/j.1398-9995.2009.01966.x 19220218

[B14] Frigerio E Pigatto PD Guzzi G Altomare G Metal sensitivity in patients with orthopaedic implants: a prospective study Contact Dermatitis 2011 64 273 279 10.1111/j.1600-0536.2011.01886.x 21480913

[B15] Jacobs JJ Skipor AK Patterson LM Hallab NJ A prospective, controlled, longitudinal study of metal release in patients undergoing primary total hip arthroplasty J Bone Joint Surg 1998 80-A 1444 1458 10.2106/00004623-199810000-00006 9801213

[B16] Jacobs JJ Skipor AK Patterson LM Hallab NJ Paprosky WG Black J Galante JO Metal release in patients who have had a primary total hip arthroplasty J Bone Joint Surg 1998 80-A 1447 1458 9801213 10.2106/00004623-199810000-00006

[B17] Urban RM Jacobs JJ Tomlinson MJ Gavrilovic J Black J Peoc’h M Dissemination of wear particles to the liver, spleen and abdominal lymph nodes of patients with hip or knee replacement J Bone Joint Surg Am 2000 82-A 457 477 10761937 10.2106/00004623-200004000-00002

[B18] Cousen PJ Gawkrodger DJ Metal allergy and second-generation metal-on-metal arthroplasties Contact Dermatitis 2012 66 55 62 10.1111/j.1600-0536.2011.01970.x 21957973

[B19] Hallab NJ Caicedo M Finnegan A Jacobs JJ Th1 type lymphocyte reactivity to metals in patients with total hip arthroplasty J Orthop Surg Res 2008 3 6 17 10.1186/1749-799X-3-6 18271968 PMC2275232

[B20] Hallab NJ Anderson S Stafford T Glant TT Jacobs JJ Lymphocyte responses in patients with total hip artroplasty J Orthop Res 2005 23 384 391 10.1016/j.orthres.2004.09.001 15734252

[B21] Hallab NJ Caicedo M Epstein R McAllister K Jacobs JJ In vitro reactivity to implant metals demonstrates a person-dependent association with both T-cell and B-cell activation J Biomed Mater Res A 2010 92 667 682 19235773 10.1002/jbm.a.32368 PMC2797558

[B22] Bohm I Brody M Bauer R Comparison of personal history with patch test results in metal allergy J Dermatol 1997 24 510 513 9301144 10.1111/j.1346-8138.1997.tb02831.x

[B23] Elves MW Wilson JN Scales JT Kemp HB Incidence of metal sensitivity in patients with total joint replacements Br Med J 1975 4 376 378 10.1136/bmj.4.5993.376 1192079 PMC1675244

[B24] Merritt K Brown SA Metal sensitivity reactions to orthopedic implants Int J Dermatol 1981 20 89 94 10.1111/j.1365-4362.1981.tb00408.x 7012051

[B25] Thyssen JP Menne T Metal allergy–a review on exposures, penetration, genetics, prevalence, and clinical implications Chem Res Toxicol 2010 23 309 318 10.1021/tx9002726 19831422

[B26] Merritt K Rodrigo JJ Immune response to synthetic materials. Sensitization of patients receiving orthopaedic implants Clin Orthop Relat Res 1996 71 79 8620661

[B27] Vermes C Chandrasekaran R Jacobs JJ Galante JO Roebuck KA Glant TT The effects of particulate wear debris, cytokines, and growth factors on the functions of MG-63 osteoblasts J Bone Joint Surg Am 2001 83 201 211 11216681 10.2106/00004623-200102000-00007

[B28] Vermes C Glant TT Hallab NJ Fritz EA Roebuck KA Jacobs JJ The potential role of the osteoblast in the development of periprosthetic osteolysis: review of in vitro osteoblast responses to wear debris, corrosion products, and cytokines and growth factors J Arthroplasty 2001 16 95 100 10.1054/arth.2001.28719 11742458

[B29] Haynes DR Crotti TN Potter AE Loric M Atkins GJ Howie DW Findlay DM The osteoclastogenic molecules RANKL and RANK are associated with periprosthetic osteolysis J Bone Joint Surg Br 2001 83 902 911 10.1302/0301-620X.83B6.10905 11521937

